# Design of Supercapacitor Electrodes Using Molecular Dynamics Simulations

**DOI:** 10.1007/s40820-018-0188-2

**Published:** 2018-01-15

**Authors:** Zheng Bo, Changwen Li, Huachao Yang, Kostya Ostrikov, Jianhua Yan, Kefa Cen

**Affiliations:** 1 State Key Laboratory of Clean Energy Utilization, College of Energy Engineering, Zhejiang University, Hangzhou, 310027 Zhejiang Province People’s Republic of China; 2 School of Chemistry, Physics and Mechanical Engineering, Queensland University of Technology, Brisbane, QLD 4000 Australia; 3 Joint CSIRO-QUT Sustainable Processes and Devices Laboratory, Lindfield, NSW 2070 Australia

**Keywords:** Electric double-layer capacitors, Molecular dynamics, Porous structure, Nanostructure

## Abstract

Electric double-layer capacitors (EDLCs) are advanced electrochemical devices for energy storage and have attracted strong interest due to their outstanding properties. Rational optimization of electrode–electrolyte interactions is of vital importance to enhance device performance for practical applications. Molecular dynamics (MD) simulations could provide theoretical guidelines for the optimal design of electrodes and the improvement of capacitive performances, e.g., energy density and power density. Here we discuss recent MD simulation studies on energy storage performance of electrode materials containing porous to nanostructures. The energy storage properties are related to the electrode structures, including electrode geometry and electrode modifications. Altering electrode geometry, i.e., pore size and surface topography, can influence EDL capacitance. We critically examine different types of electrode modifications, such as altering the arrangement of carbon atoms, doping heteroatoms and defects, which can change the quantum capacitance. The enhancement of power density can be achieved by the intensified ion dynamics and shortened ion pathway. Rational control of the electrode morphology helps improve the ion dynamics by decreasing the ion diffusion pathway. Tuning the surface properties (e.g., the affinity between the electrode and the ions) can affect the ion-packing phenomena. Our critical analysis helps enhance the energy and power densities of EDLCs by modulating the corresponding electrode structures and surface properties.
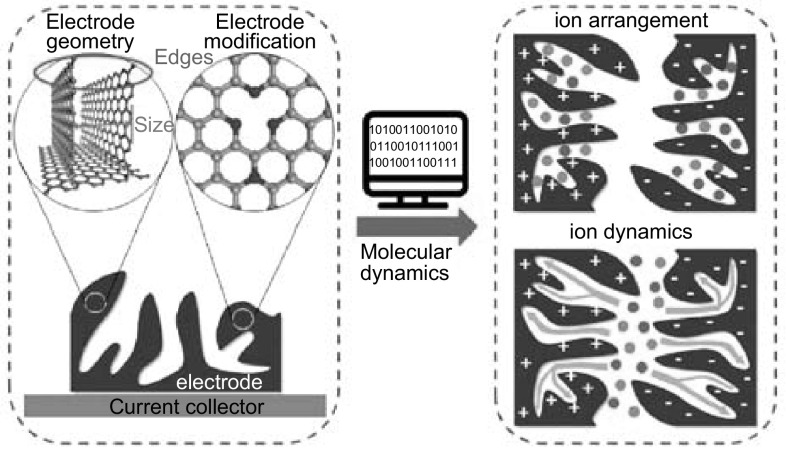

## Highlights

- Capacitive behaviors of electric double-layer capacitors (EDLCs) are strongly related to electrode geometry and electrode modification.
- Molecular dynamics (MD) studies on EDLCs’ performances of electrode materials from porous to nanostructures are summarized.
- MD could provide guidelines for the optimum design and fabrication of active materials.

## Introduction

Electric double-layer capacitors (EDLCs) are capacitive or non-faradaic energy storage devices, featuring high power delivery and rapid charge–discharge cycles [1, 2]. They occupy an intermediate position between dielectric capacitors and batteries and are versatile enough as energy storage systems, stand-alone or combined with batteries [3]. Owing to higher power density and longer cycle life than those of batteries, as well as superior energy density than that of dielectric capacitors [4, 5], EDLCs are best suitable for occasions when fast charge/discharge rates and reliability are needed, such as energy recovery devices [6], uninterruptible power systems [7], buffers for power grid [8], high-performance filter capacitors [9]. The performances of EDLCs are characterized by the energy density, power density, rate performance, cycle stability, leakage current, open circuit voltage, safety and cost [10].

EDLCs are comprised of two oppositely charged electrodes separated by electrolyte. The electrostatic interaction between the electrified electrode and electrolyte ions leads to the physical adsorption of counterions and desorption of co-ions at the electrode/electrolyte interfaces [10]. This process leads to the ion separation and charge accumulation, thus causing the capacitive effect [1]. For industrial and large-scale application of EDLCs, rational optimization of electrode–electrolyte interactions is of vital importance to enhance device performances. One of the effective methods is customizing the electrode materials, e.g., engineering pore geometry/size and doping defects/functional groups [11, 12]. The suitable materials should be abundant and cheap, having large specific surface areas and excellent conductivity, non-toxic and environment-friendly, and easily processable [13, 14]. This is why carbon materials are among the most promising electrode materials [3, 13].

The choice of electrode materials is closely related to EDL structure. Surface topography and modification of electrodes affect ion accumulation at the electrode/electrolyte interface. For example, a higher surface curvature of carbon nanotubes is beneficial for a greater population of ions at the interface [15]. Within the carbon nanotube micropores, a single-file distribution is recognized near the high curvature surface [16]. The sharp edges of graphene nanostructures tend to aggregate more electrons, obviously boosting the separation of counterions and co-ions within the EDL-dominant area [17, 18]. The EDL structure has been studied since 1879 [19], in which an original but crude Helmholtz model was proposed. This theoretical model of Helmholtz was used to elucidate the separation of two layers with the opposite charges and the formation of EDL at the interface of solid/liquid [19], similar to those in conventional dielectric capacitors. Subsequently, the Helmholtz model was further improved by Gouy in 1910 [20] and Chapman in 1913 [21]. These models incorporated the continuous distribution and motion of electrolyte ions. This improved model is referred to as Gouy–Chapman model, where the potential in EDL decreases exponentially with distance to the electrode surface [22]. With the combination of the Helmholtz model and the Gouy–Chapman model, Stern recognized two areas featuring distinct ion distributions, namely the inner and outer layers [10, 23]. The accumulation of densely packed ions occurred in the inner layer (also called compact layer or Stern layer), while the hydrodynamic motion of ions exists in the outer layer (diffuse layer) [22]. Nowadays, these traditional theories could serve as a guideline for optimization of EDLCs, especially suitable for mesoporous materials.

As new nanomaterials are developed, new challenges emerge. New effects, closely correlated with the nanomaterials, include anomalous increase in capacitance [11, 24–28], desolvation [25, 29–31], surface roughness [32–34], doping effect [35–39] and edge effect [17, 18, 40]. The anomalous increase in capacitance and desolvation effect are intrinsic for sub-nanometer pores. Owing to these new effects, the arrangement of ions at the interface is heterogeneous, obviously different with that predicted by EDL theoretical models. Therefore, the solutions for these challenges should be based on experimental methods and numerical simulations.

On the one hand, electrolyte structuring in nanoconfinement is measured by some experimental techniques, including nuclear magnetic resonance (NMR) [41, 42] and electrochemical quartz crystal microbalance (EQCM) [43, 44]. NMR acquires the information about dynamic behavior of ions at nanoscale, including the distribution and dynamics of electrolyte ions [45]. EQCM is a sensitive tool based on in situ gravimetric detection to investigate the ion transport from the bulk to the pores in electrochemical systems. The principle is to measure the variation of its resonance frequency, which is converted into mass variation when imposing an alternating electric field [46]. Both of these techniques have been employed to study the adsorption mechanism of ions inside traditional porous structures, such as activated carbon [42, 43, 47] and carbide-derived carbon [46, 48]. Importantly, the use of these techniques is extended to novel nanomaterials. Recently, Li et al. [45] employed solid-state NMR to investigate the adsorption of BF_4_^−^ ions inside a graphene film, a typical 2D nanomaterial. In addition, net change of mass in zeolite-templated carbon has been experimentally monitored using EQCM [49]. Employing the NMR techniques, Zhang et al. [50] confirmed the existence of various oxygen-contained functional groups and two kinds of water molecules within the graphene oxide films. Using the EQCM tests, Barisci et al. [51] successfully monitored the mass change of different electrolytes within the single-walled carbon nanotube films.

On the other hand, numerical simulation has been one of the most powerful tools for investigating the physical and chemical phenomena. Different simulation methods have been employed to characterize the capacitive behaviors of EDLCs, such as density functional theory (DFT), finite element method (FEM) and molecular dynamics (MD) simulation. DFT calculations are hard to be extended to a realistic representation of the porous electrodes or complex ion structures owing to the huge computational costs. Besides, FEM calculations, generally described by a continuum model (e.g., Nernst–Plank equations), commonly fail to capture the capacitive behaviors of nanoporous electrodes with densely packed electrolytes (e.g., ionic liquids). In particular, MD is an effective simulation method to describe the interaction between particles and reproducing their dynamical behaviors in nanoscale physical phenomena. In the MD system, physical motions of atoms and molecules are characterized by solving Newton’s equations of motion with the help of numerical solutions, e.g., Verlet method. The forces imposed on the particles are calculated according to the specified interatomic potentials or force fields. Iterations between calculating the force and obtaining the motion of particles reveal the detailed information about their motions and trajectories for the entire process. By tracing the trajectories of particles, MD simulations can deliver the whole picture for dynamic evolution, obtaining the macroscopic characteristics for the simulation system. For EDLCs, through specifying the interactions between ions and ion pathways, one can obtain the distributions and motions of electrolyte ions, reflecting their energy storage and dynamics behavior, respectively. Due to its principle, MD simulation provides the insights at atomic level into the mechanisms of charge storage and ion transport, advancing our understanding and helping enhance the electrocapacitive performances of EDLCs based on nanomaterials. MD is a precise method to investigate complex phenomena and challenges existing in nanomaterials. Besides, MD modeling can also be applicable to many new materials (e.g., MoS_2_) [52–54]. However, MD simulations are still challenging to capture the reaction or conversion process of pseudocapacitance, owing to the lack of accurate reactive force field potentials. This review aims at revealing the mechanisms of energy storage and ion dynamics with the aid of MD simulations and to critically examine the dynamics of energy storage inside electrode materials with various structures and morphology ranging from porous to nanostructures.

## Molecular Dynamics Investigations of Porous Materials

Porous carbons, typically activated carbon (AC) and carbide-derived carbon (CDC), are carbon matrixes or frames containing polyhedral voids, also known as ‘foam’ materials. Their typical features are porosity as well as the existence of numerous corrugated and curved pores. Due to the large specific surface areas, broad ranges of operating temperature and earth abundance, these materials have attracted attention for utilization in EDLCs [55]. Porous carbons contain a large fraction of amorphous carbons characterized by diverse pore size distributions, shapes and structures.

### Activated Carbon

AC exhibits a broad pore size distribution, categorized into micropores (< 2 nm), mesopores (2–50 nm) and macropores (> 50 nm), with a large number of interconnected pores [10, 56, 57]. AC is synthesized through physical or chemical processing of carbonaceous precursors, resulting in opening additional pores and increased porosity [58]. By altering carbonaceous precursors and synthesis conditions, their structures can be controlled to a certain extent, e.g., porosity, distribution of pore size and amount of interconnected pores [3, 22]. Recently, AC has been one of the most widely used porous materials in commercial applications as electrodes of supercapacitors, due to its moderate cost and specific surface area of 3000 m^2^ g^−1^ [10]. However, few MD simulation investigations on AC have been carried out. The reason is that it is hard to construct a representative numerical model for AC, attributed to its complex structure. Coconut shell AC (CSAC) is a special case and most of the pores are micropores, which is a suitable range of pore size for MD simulations. To construct a numerical model with random structure of CSAC, a suitable method for numerical generation should be chosen when considering its feasibility. Direct construction of numerical models can only produce some regular structures, which is far from reality. Quenched MD can simulate the process of thermal quenching to obtain an amorphous carbon structure, but its initial approximate structure should be known. Reverse Monte Carlo (RMC) protocol, proposed by Mcgreevy et al. [59], can directly produce the amorphous structure according to its experimental structural data, e.g., radial distribution function and diffraction data, without considering its initial state. The realistic model of CSAC was built by Pikunic et al. with the help of RMC protocol [60]. This model is applied to generate several microporous carbon models featuring atom configuration that are almost identical to the experimental structural data [60]. The RMC protocol is carried out in the following sequence: (1) Initial configuration. Randomly create a three-dimensional array whose density is equal to the target one. (2) Random and tiny movement of a single particle to minimize the deviation between the obtained structural data and the expected one. (3) Repeat the process until the deviation oscillates around the equilibrium and the expected structure is obtained.

**Fig. 1:**
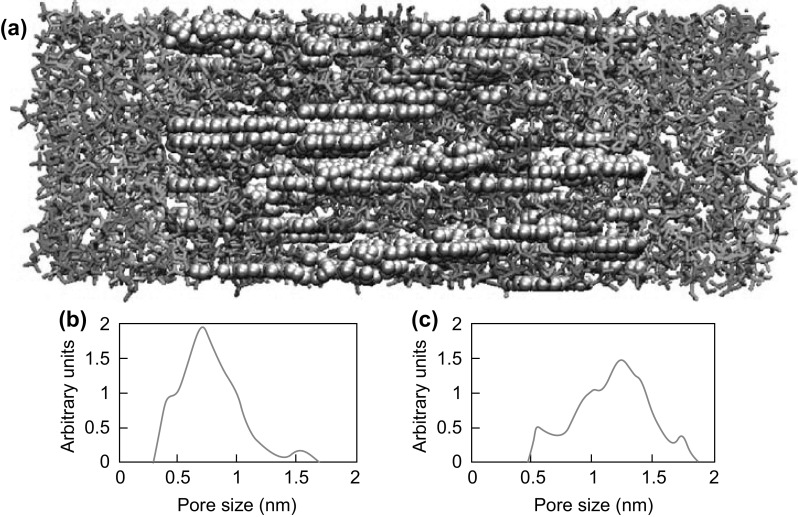
**a** CSAC model and the pore size distributions for average pore size of **b** 0.75 nm and **c** 1.23 nm (gray: carbon atoms, red: cations and green: anions). Reprinted with permission from Ref. [61]. Copyright (2014) American Chemical Society. (Color figure online)

The obtained structure is then used to construct a numerical electrode/electrolyte model for MD simulations. Through investigating the motions of molecules and ions inside the electrode/electrolyte model, the obtained results could reveal the effect of structural characteristics on device performance. Rajput et al. [61] employed the RMC protocol to construct a CSAC numerical model and to compare this model with the electrode material with homogeneous pore size. This approach helped elucidate the key role of the heterogeneous pore size and shape, as well as the complex morphology. The obtained CSAC model is shown in Fig. 1a, and the pore size distributions for the two kinds of average pore size are shown in Fig. 1b, c, respectively. They found that the heterogeneity of pore size distribution and existence of interconnected pores could weaken the confinement effect originating from pore walls. In this case, the behavior of ions inside the CSAC model is similar to the behavior in a bulk electrolyte, in terms of the distribution and dynamics.

### Carbide-Derived Carbon

In contrast to AC, CDC is a product derived from diverse carbides removing their atoms except carbon, which possesses a narrow and controllable pore size distribution [62]. By tuning chlorination temperature and carbide precursors, the average pore size of CDC could reach several nanometers, or even several Ångstroms [62–64]. Using CDC as an active electrode material, Chmiola et al. [11] in 2006 found the anomalous change of capacitance as its average pore size became less than 1 nm. This anomalous phenomenon challenges the traditional view that pores whose sizes are less than the diameter of solvated electrolyte ions, cannot contribute to energy storage. This amazing finding caused the widespread concern of researchers, as well as led to enormous studies on the interfacial structure of EDL and the charge storage dynamics in CDC [27, 31, 65, 66]. Since Palmer et al. [65] employed quenched molecular dynamics to construct a realistic model of CDC in 2010, the simulation work on CDC has intensified, mainly focusing on energy storage and charging rates [27, 31, 66, 67].

**Fig. 2:**
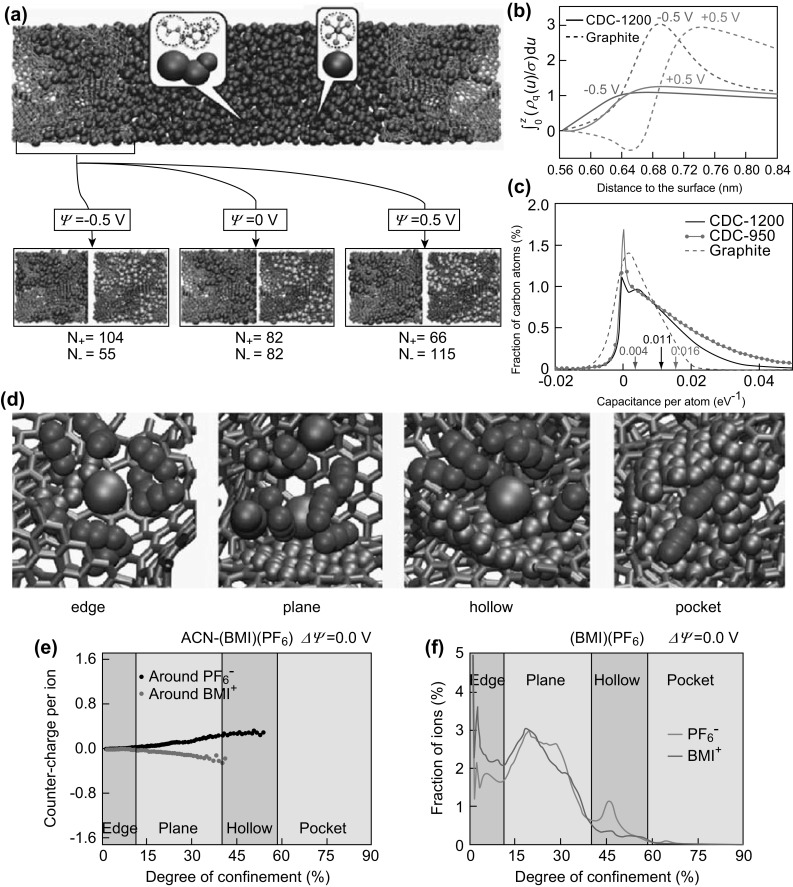
The energy storage inside CDC electrode. **a** The snapshot of CDC model and quantitative picture of ions adsorbed into the model under different potentials (blue: carbon atoms, red: BMI^+^ ions and green: ${\text{PF}}_{6}^{ - }$ ions). **b** Integral of charge density over the distance to surface for CDC-1200 and graphite. **c** The distributions of capacitance per atom for three kinds of carbon material, and the average value is indicated by the arrow. Reprinted with permission from Ref. [27]. Copyright (2012) Nature Publishing Group. **d** Four typical structures of adsorption location (edge, plane, hollow and pocket) with the different DoC. The variation of **e** fraction of ions and **f** countercharge per ion with DoC. Reprinted with permission from Ref. [31]. Copyright (2013) Nature Publishing Group. (Color figure online)

On the one hand, sub-nanometer pores in CDC could enhance its capacitance. Since the discovery of the anomalous increase in capacitance, it should be noticed that a higher capacitance not only is ascribed to a larger specific surface area, but also is due to the presence of sub-nanometer pores. The introduction of sub-nanometer pores could improve the efficiency of charge storage, mainly ascribed to two structural factors (i.e., pore size and microstructure). To explain the high capacitance for CDC, Merlet et al. [27] were first to quantitatively describe the phenomenon of room-temperature ionic liquid (RTIL) ions located in a numerically constructed CDC structure with the help of MD simulations, as shown in Fig. 2a. The capacitance value obtained from their simulation work (125 F g^−1^) is coincident with the experimental results and is higher compared to simple planar structures. They attributed the low capacitance for planar graphite to the emergence of several adsorbed layers with damped oscillations and called this observation an overscreening effect. The charge accumulated in the first adsorbed layer is larger than the electrode charge, but is counterbalanced by the next layer, leading to a relatively small fraction of adsorbed ions utilized for energy storage [27, 68]. In contrast, the high capacitance for CDC is due to the reduced overscreening, as shown in Fig. 2b. There is only one adsorbed layer, whose charge balances exactly with the electrode charge, leading to the high storage efficiency. The enhancement in capacitance is also reflected by the distribution of charge on carbon atoms in their work, as shown in Fig. 2c. The calculated average value of capacitance for CDC is higher compared to graphite.

The microstructure of sub-nanometer pores can affect the dynamics of energy storage. Merlet et al. [31] studied the confinement effect for four types of adsorption locations on the edge, planar, hollow and pocket sites, as shown in Fig. 2d. They found an increase in desolvation and stored charge with the increasing degree of confinement (DoC). The solvated ions must be partially stripped of their hydration shells to enter the narrow pores in CDCs, inducing more charges accommodated inside highly confined pores. The charge could be more effectively stored at the sites with higher confinement, according to Fig. 2e [31]. However, most of the ions reside in the edge and plane sites, while some are adsorbed into the hollow and pocket sites, as shown in Fig. 2f. Therefore, it is important to develop some feasible methods to drive more ions to enter the sites with strong confinement, for example by imposing charge on the electrode and by removing solvents. The typical structures of sub-nanometer pores are categorized into convex and concave structures, with the higher influence of radius on capacitance for convex structure [69]. To characterize the structure of nanoporous carbon, Pak et al. [70] introduced the pore shape factor (PSF) and studied the variation of capacitance with PSF, which agrees well with the experimental results.

**Fig. 3:**
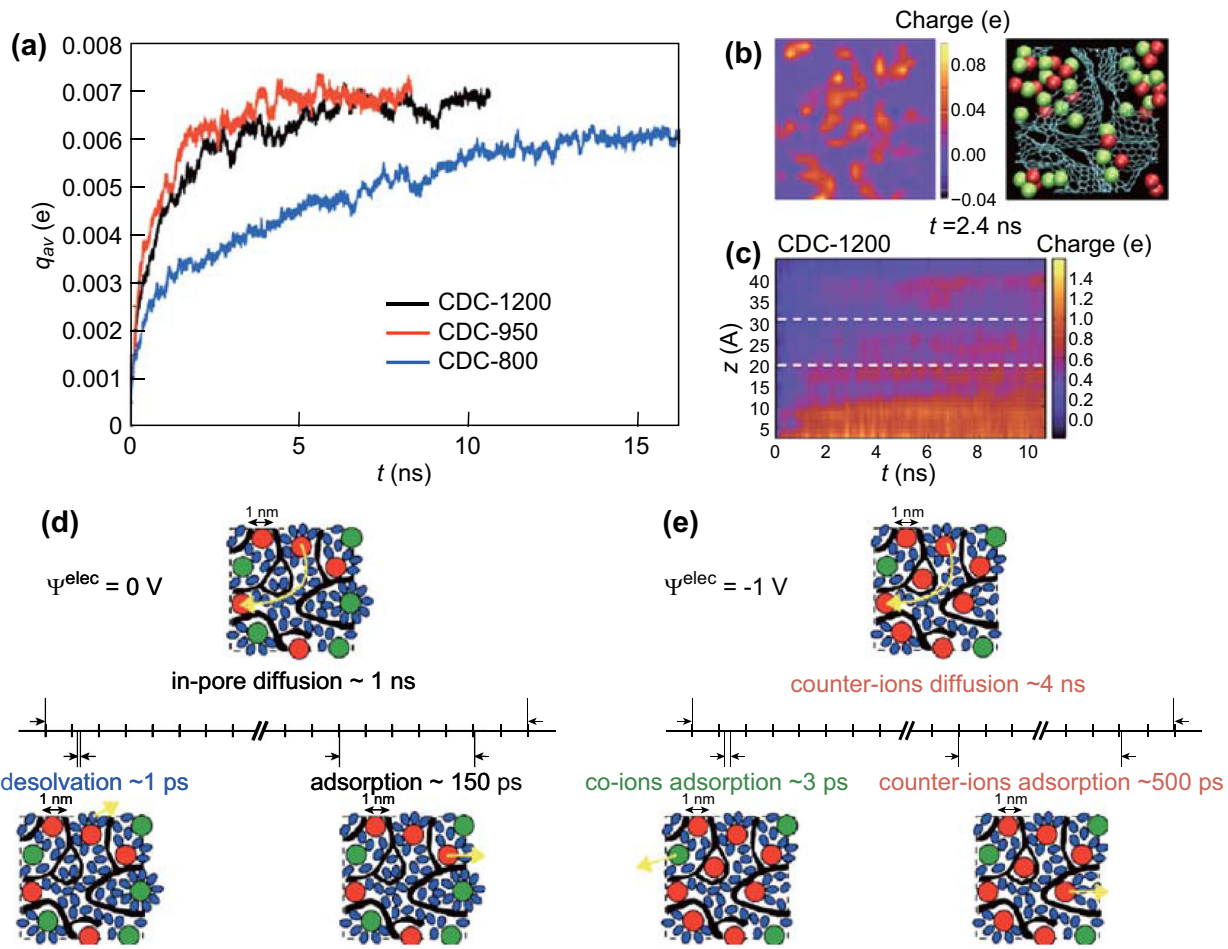
Charging dynamics and characteristics times of each stage in CDC electrode. **a** Time evolution of average charge per atom for CDC-800, CDC-950 and CDC-1200. **b** The local charge (left) and instantaneous ionic position (right) for CDC-1200 when *t* = 2.4 ns. **c** Evolution of local charge with time for CDC-1200. Reprinted with permission from Ref. [67]. Copyright (2014) American Chemical Society. Summary of various characteristics times for three stages when ions approaching the surface at **d** 0 V and **e** − 1 V. Reprinted with permission from Ref. [66]. Copyright (2015) American Chemical Society

On the other hand, the size and heterogeneous structure of sub-nanometer pores in CDC exert a tremendous influence on the ion dynamics and charging rate. According to the analysis from Ragone plot, the high energy density, achieved when sub-nanometer pores are present, is always accompanied with the low power density. The power density of the systems has a close relationship with ion transport inside the pores. Therefore, addressing the dynamic aspects should be focused on investigating the motion of electrolyte ions in EDLCs in the process of charge/discharge. The relation between the ion transport and charging rate at a macroscale could be established by using a transmission line model and the equivalent circuit model. Based on these methods, Péan et al. [67] compared the dynamic charging processes for three kinds of CDC microstructures with different average pore sizes. Their work showed that charging time decreases with the increase in the average pore size (Fig. 3a), despite the differences in the pore structure and connectivity. Moreover, the heterogeneous structure of CDC leads to heterogeneous charging. Figure 3b depicts the instantaneous and heterogeneous distribution of charge on a local region at 2.4 ns [67]. The evolution of charge with time (Fig. 3c) indicates the distinct charging dynamics for different layers of CDC.

The comparison of characteristic times for different stages is crucial for further enhancement of the power density. In the process of ion transport into electrified nanopores from the bulk, there could be three typical stages, including desolvation, diffusion and electrosorption. Péan et al. [66] investigated the time hierarchy for the three stages when the electrode potentials are 0 and −1 V. For the case of a neutral electrode (Fig. 3d), their results indicated that the time for desolvation is much shorter than the time for the adsorption. On the other hand, the time for adsorption is much shorter than the time for in-pore diffusion. Comparing the hierarchies for the neutral electrode (Fig. 3d) and the electrified electrode (Fig. 3e), all three characteristic times for the neutral electrode appear to be shorter than the case of electrified electrode.

Another CDC numerical model is platelet model consisted by numerous carbon platelets with a random distribution [71]. It has a high computational efficiency, but is less realistic compared to quenched MD. One-electrode model can help minimize the computational cost, with few differences with two-electrode model in simulation results [72]. To validate the methodology, Merlet et al. [73] compared two different methods for mimicking the polarization of electrode, i.e., fixed charge method (FCM) and constant potential method (CPM), with the latter being a more realistic method. This confirms the important role of CPM, although the cost of computational resources is high. Some simulation works have been implemented on the reliability of FCM and the improvement of CPM [74, 75].

## Molecular Dynamics Investigations of Two-Dimensional Nanomaterials

As the dimension of electrode materials becomes close to several nanometers, exotic energy storage mechanisms emerge for diverse nanomaterials. Two-dimensional (2D) nanomaterials, typically graphene, are made of one or several graphitic monolayers without defects, each monolayer containing hexagonal carbon atom rings [76–78]. Graphene was discovered by Novoselov and Geim et al. [79] and has many outstanding properties. Monolayer graphene has a theoretical specific area of ~ 2630 m^2^ g^−1^ [80], twice as that of single-walled carbon nanotubes [77], and EDL capacitance of ~ 21 μF cm^−2^ (the converted gravimetric specific capacitance is ~ 500 F g^−1^) [58, 81]. Graphene also exhibits exceptionally electrical conductivity, especially along the basal plane, which promotes ion motion and electron propagation in the process of charge/discharge [77]. Additionally, superior mechanical properties, e.g., strength and flexibility, improve the lifetime of graphene-based EDLCs [77, 82].

**Fig. 4:**
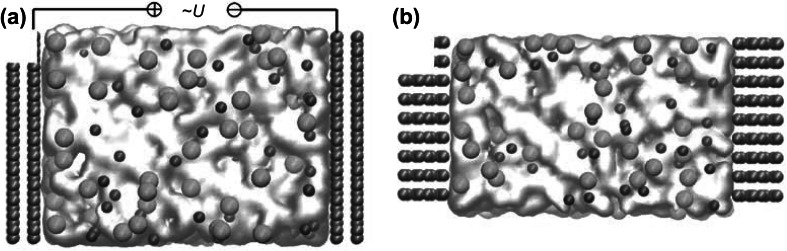
Numerical structures for **a** planar model and **b** slit-type model. Reprinted with permission from Ref. [18]. Copyright (2016) American Chemical Society

Owing to its unique structure and superb properties, graphene receives widespread attention and currently becomes one of the most commonly utilized materials for electrochemical energy storage. Controlling surface morphology and designing pore structure in graphene have a significant effect on the energy and power density of EDLCs. By constructing the electrode model made of graphene, MD simulation could provide a theoretical guidance for the supercapacitor electrode design. Two typical numerical models for mimicking graphene electrodes are proposed, namely a planar model and a slit-type model. The planar model is described as one region of electrolyte enclosed by two oppositely charged parallel plates separated by about several or dozens of nanometers, as shown in Fig. 4a. The slit-type model has two face-to-face electrodes with the opposite charge, as illustrated in Fig. 4b. For each side, the electrode is made of two or more parallel plates with the same sign of charge. Slit pores, composed of parallel graphene layers, are developed within the stacked graphene networks. Compared with the numerical model of CDC, the major feature of the graphene model is its homogeneous pore size, without considering the complex pore structure.

### Planar Model

The planar model is widely used as a pristine model for direct characterization of monolayer graphene, revealing and describing the fundamental mechanisms of arrangement and motion of electrolyte ions. Modification of the electrode morphology could significantly affect the EDL structure and properties. Recent simulation progress based on planar model is discussed here, including surface topography and doping effect.

#### Surface Topography

Realistic graphene structures should contain the basal plane and the edge plane. While the basal plane is atomically smooth, the edge plane is corrugated at atomic scale. The corrugated plane needs a significant number of parameters to characterize their appearance. For instance, surface roughness strongly affects energy storage and charge distribution and complicates MD simulations.

**Fig. 5:**
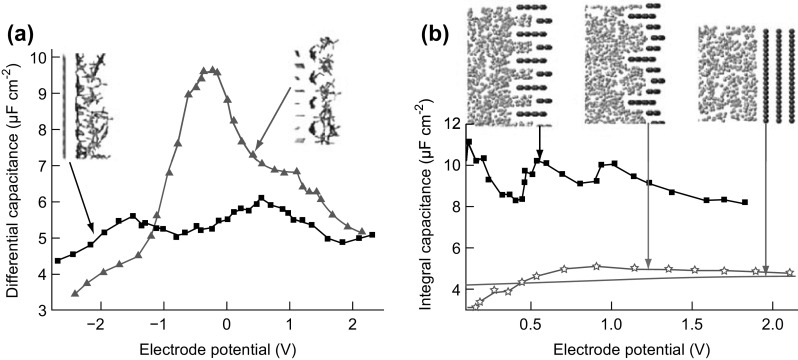
Differential capacitance as a function of electrode potential. For **a** flat and rough surface. Reprinted with permission from Ref. [32]. Copyright (2011) American Chemical Society. For **b** the surface with different surface nanopatternings. Reprinted with permission from Ref. [33]. Copyright (2012) American Chemical Society

On the one hand, surface roughness could improve the electric charge storage. The principle of surface roughness is to generate sub-nanometer pores or increase the pore numbers on the surface. Originating from these sub-nanometer pores, the main difference between the flat and corrugated surfaces is the confinement effect unique for corrugated surfaces. Therefore, corrugated surfaces have a higher efficiency of charge storage than flat surfaces. As illustrated by Vatamanu et al. [32], a higher capacitance is observed for atomically corrugated surfaces than for flat surfaces for nearly all electrode potentials, as shown in Fig. 5a. In particular, the capacitance of a corrugated surface is almost twice as high compared to the flat one near the potential of zero charge (PZC). In addition, rational design of surface roughness could maximize the energy storage behavior. Xing et al. [33] made a comparison between the atomically flat surface and two corrugated surfaces with varying degrees of nanopatterning, as shown in Fig. 5b. A significant increase in capacitance was observed for the corrugated surface with a small groove width, while the capacitance for the corrugated surface with large groove width was found approximately equal to that of flat surface. The highest efficiency of energy storage is when the groove width approaches the diameter of electrolyte ions. However, improper surface roughness could lead to a small increase in capacitance, or even be detrimental to the energy density.

On the other hand, surface roughness could change the relationship between the capacitance and the electric potential. The above relationship could be represented by the dependence of capacitance on voltage. The shape of *C*–*V* curve is a significant factor to quantify the effect of the surface roughness. For a flat surface, the shape of the *C*–*V* curve is relatively flat (Fig. 5a) [32], due to its easy accessibility for electrolyte ions. However, surface roughness could induce a stronger dependence of the capacitance on the applied potential, i.e., a more rapid decrease in capacitance generally. Tuning the surface roughness would affect the dependence of capacitance versus the electrode potential. A stronger dependence of the capacitance was detected for the case of a small groove width, in contrast to the weaker dependence for a larger groove width (Fig. 5b) [33].

However, there are conflicting data about the shapes of *C*–*V* curves for flat and corrugated surfaces. Simulation results suggested that the *C*–*V* curves were camel-shaped for RTIL ions near the atomically flat surface, while a bell-shaped *C*–*V* curve was recognized for the corrugated cases [32]. Another work performed by the same author demonstrated a camel-shaped differential capacitance for the both of the above surface types [38]. In addition, by using the theoretical analysis, Kornyshev [83] discovered that the shape of a *C*–*V* curve for an ideal flat surface is determined by the electrolyte compressibility. Nevertheless, different shapes of *C*–*V* curves for graphene surfaces with different morphologies cannot be well interpreted, so its underlying mechanism needs further investigation.

The effects of temperature [84] and ion types [38, 85, 86] on the capacitance and shapes of *C*–*V* curve for corrugated surfaces have also been observed. The effect of the potential and temperature on the capacitance appears to be much stronger for the corrugated surfaces, as Vatamanu et al.’s work indicated [84]. Moreover, to investigate the influence of the ion type, a series of works has been carried out. The first work compared two RTILs, i.e., [BMIM][BF_4_] and [BMIM][PF_6_], and found that a higher asymmetry could result in a larger difference between the maximum and the minimum of capacitances [86]. The second work revealed that RTILs with FSI or with TFSI anions feature different variation trends for the shape of *C*–*V* curve [85]. A camel-shaped curve is observed for TFSI^−^ ions, while the shape of *C*–*V* curve makes a transition from bell to camel for FSI^−^ ions. Apart from the studies on anions, a work concerning cations was also carried out [38]. Their work confirmed that the increase in chain length of cations could lead to the slight reduction in capacitance [38].

#### Doping Effect

Doping effect is referred to as the introduction of heteroatoms or functional groups to the structure of a carbon monolayer. Doping heteroatoms, typically nitrogen atoms, could change the structure of a hexagonal ring where carbon atoms are replaced by heteroatoms. However, doped functional groups, typically oxygen-containing groups, only substitute hydrogen atoms on the surface. Due to this significant difference, the former case requires a combination of density functional theory (DFT) and MD, while the latter case could be simulated by a single MD technique. DFT calculation represents a successful approach to simplify complex electron–electron interactions into an effective one-electron potential [87]. This method is used to calculate the atomic and electronic structures of diverse materials. By analyzing their atomic and electronic structures, some critical parameters could be obtained in the process of energy storage, e.g., capacitance in electrode (i.e., quantum capacitance) and the accurate partial charge distribution on the doped electrode. Based on MD and DFT methods, the investigations on the doping effect of electrodes are discussed below.

Doping nitrogen atoms could increase the quantum capacitance and overcome the limitations of total capacitance at PZC. For the pristine graphene, its quantum capacitance is nearly zero at PZC, leading to the extremely low value of total capacitance, according to the series connection of EDL capacitance and quantum capacitance. To avoid this limitation, one of the feasible methods is doping with nitrogen atoms. This method can increase the quantum capacitance, by changing the electrode structure. The experimental result shows a substantial increase in capacitance for nitrogen-doped graphene, whose specific capacitance is about 280 F g^−1^ (~ 70 F g^−1^ for pristine graphene) [39].

**Fig. 6:**
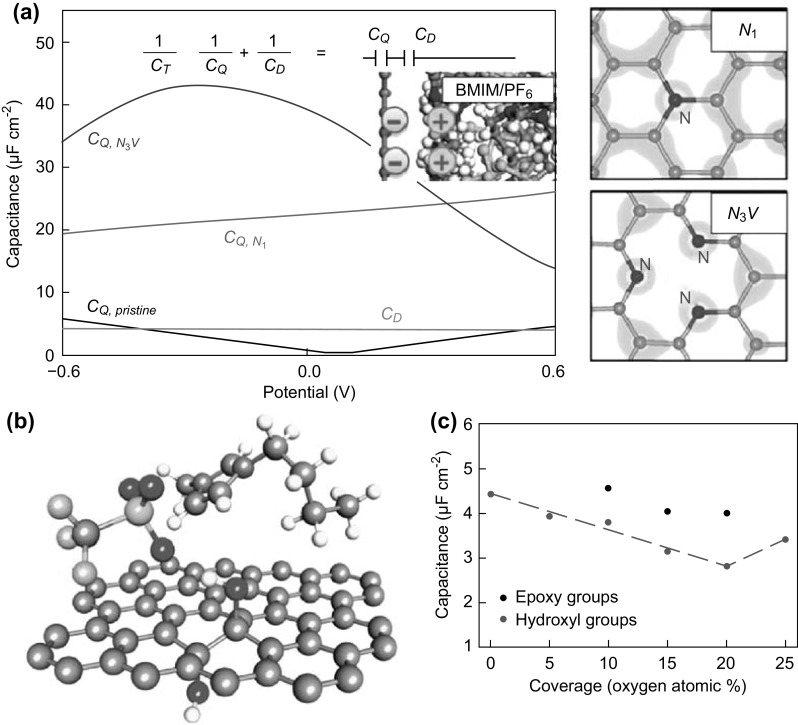
Doping heteroatoms and functional groups. **a** The diagram of two kinds of doping nitrogen atoms and the variation of EDL capacitance and quantum capacitance with potential. Reprinted with permission from Ref. [35]. Copyright (2013) American Chemical Society. **b** The diagram of doping oxygen-containing functional groups. **c** The capacitance as a function of oxidation ratio for epoxy and hydroxyl groups. Reprinted with permission from Ref. [36]. Copyright (2014) American Chemical Society

For the sake of analyzing the effect of doping nitrogen atoms at atomic scale, a hybrid method mentioned in the previous section was utilized. Paek et al. [35] performed a simulation research for two types of nitrogen-doped structures, namely N_1_ and N_3_V (Fig. 6a), and proved that their total capacitances were much higher compared to the pristine graphene layer. As shown in Fig. 6a, the high total capacitance is mainly due to the increase in quantum capacitance, while the EDL capacitance remains almost equal for all the three cases. Comparing the quantum capacitances for N_1_ and N_3_V, it is clear that the quantum capacitance of N_3_V structure is not always larger compared to N_1_ structure. Another suitable heteroatom is boron, which plays a similar role as the nitrogen atom. An enhancement of quantum capacitance for boron-doped graphene was reported by Vatamanu et al. [88]. Nitrogen/sulfur co-doped graphene has some superiorities compared to nitrogen-doped graphene, e.g., the higher quantum capacitance at the suitable voltage (~ 60 μF cm^−2^ for nitrogen-doped graphene and ~ 100 μF cm^−2^ for co-doped graphene) [89]. Based on the above findings, it would be valuable to control the structure of nitrogen-doped graphene as well as co-doped graphene, when considering the optimum operating potential.

Doping oxygen-containing functional groups is detrimental for the EDL capacitances and structures. Hydroxyl and epoxy groups are the most common oxygen functional groups in graphene layer, generated in the process of oxidizing of graphene layer. However, these groups could not be completely removed during the reduction of graphite oxide (GO) [90]. The existence of oxygen atoms on graphene surface could not only change the conductivity of electrode, but also modify the affinity between the electrolyte ions and the electrode surface. On the one hand, the variation of affinity can attract or repel more ions, leading to the changes in EDL structures and, more importantly, the effectiveness of energy storage.

A diagram of doping oxygen-containing functional groups is shown in Fig. 6b. When graphene surface is doped with oxygen-containing functional groups, the total capacitance tends to decrease with the increasing concentration of hydroxyl and epoxy groups, apart from pseudocapacitive effect, as shown in Fig. 6c [91]. However, there exists a contradiction about the comparison between the effects of hydroxyl and epoxy on the decreasing capacitance. According to Kerisit et al.’s work [36], hydroxyl groups lead to faster decrease in capacitance than in the case of epoxy groups. However, Xu et al.’s work [92] indicated that the influences of both for hydroxyl and epoxy are similar. On the other hand, doping oxygen-containing functional groups could lead to the reduction in the conductivity of electrode. The effects of oxygen-containing groups on electrode capacitance require further studies.

The characteristics of RTIL ions play an important role in EDL capacitance. In general, smaller size of RTIL ions could result in a higher capacitance for corresponding electrode (i.e., cation for cathode and anion for anode) [38, 85, 93–95]. However, a simulation work indicated that the size of anions leads to a significant increase in the capacitance of cathode [96]. For electrolyte ions with different shapes, the capacitance is affected by the ion sizes as well as ion shapes [72], e.g., the almost identical *C*–*V* curves for four types of amino acid ILs [97]. In addition, a few works have been carried out about the influence of chain length on the ion dynamics [98] and thermal stability [99]. Recent simulation and experimental studies suggested that the superconcentrated aqueous electrolytes could also optimize the energy storage performances of EDLCs, e.g., enhancing the electrochemical stability and broadening the voltage window [100, 101].

### Slit-Type Model

Since Chmiola et al. [24, 25, 102] first discovered the anomalous increase in capacitance inside the sub-nanometer pores, size effect, i.e., confinement effect, in graphene-based electrodes has also received a significant attention. For investigations into the confinement effect, a slit-type model is used. Compared with the planar model, the slit-type model based on multilayer graphene presents more information about the energy storage. Recent simulation work based on the slit-type model is mainly focused on the interlayer spacing of graphene (confinement effect) and edges of graphene (edge effect), as discussed below.

#### Confinement Effect

In contrast to the confinement effect originated from CDC, the confinement in graphene is quite different, mainly reflected in the homogeneous pore size distribution and structure. This feature helps directly reveal the confinement effect in sub-nanometer pores, excluding the interference from heterogeneous structure. The confinement effect in the slit pores could give rise to several exotic phenomena, including the abnormal behavior of charge storage and restricted dynamic characteristics.

**Fig. 7:**
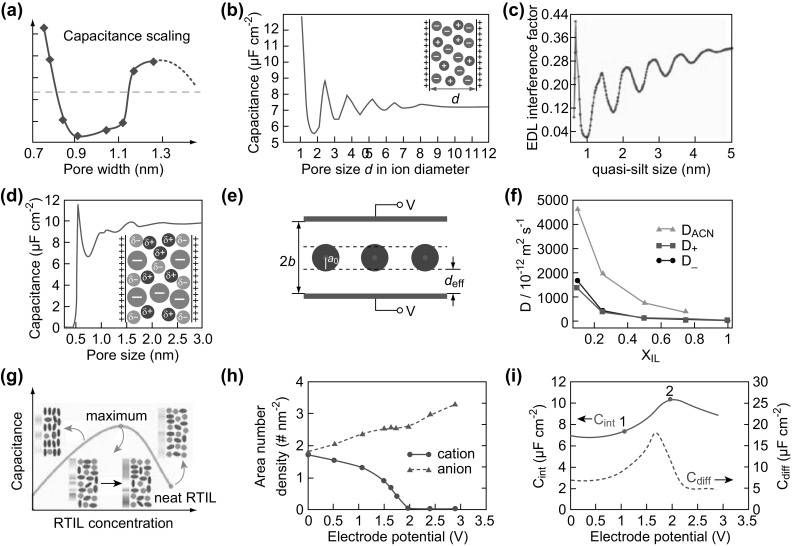
The capacitance and number density in slit-type models. The capacitance as a function of pore size. For **a** small range. Reprinted with permission from Ref. [24]. Copyright (2011) American Chemical Society. For **b** large range. Reprinted with permission from Ref. [26]. Copyright (2011) American Chemical Society. **c** The variation of EDL interference factor with the pore size. Reprinted with permission from Ref. [103]. Copyright (2011) American Chemical Society. **d** The variation of capacitance when adding organic solvents. Reprinted with permission from Ref. [106]. Copyright (2012) American Chemical Society. **e** The diagram of a sandwich capacitance model. Reprinted with permission from Ref. [25]. Copyright (2010) American Chemical Society. Adding ACN molecules, the variation of **f** self-diffusion coefficient. Reprinted with permission from Ref. [109]. Copyright (2016) American Chemical Society. The variation of **g** capacitance with ACN concentration. Reprinted with permission from Ref. [108]. Copyright (2016) American Chemical Society. At different voltages, **h** capacitance and **i** number density. Reprinted with permission from Ref. [113]. Copyright (2012) American Chemical Society

First, the confinement effect leads to the oscillatory behavior of capacitance. The early observation, an anomalous increase in capacitance, was discovered by experimental methods and has stimulated further research. This abnormal phenomenon becomes obvious as the pore width approaches the diameter of electrolyte ions. However, for the middle pore size (i.e., one or several times of the ion diameter), their variation of capacitance needs further study. A series of simulation works has been carried out concerning the behavior of charge storage for nanopores and mesopores. These works advanced our understanding of the mechanisms of variation of capacitance. Beside the anomalous increase in capacitance, Wu et al. discovered a further increase in capacitance when the pore size was slightly increased beyond 1.1 nm (Fig. 7a) [24]. Inspired by this new finding, the oscillatory behavior of capacitance versus the pore size could be noticed. Jiang et al. [26] and Feng et al. [103], respectively, found an oscillatory behavior for capacitance with the increase in the pore size, as shown in Fig. 7b. They attributed the oscillatory behavior to the interference from EDLs. The capacitance is extremely sensitive to the variation of pore size for the sub-nanometer pores, but is less affected for the mesopores. To predict the oscillatory behavior of *C*–*V* curve, Feng et al. [103] introduced the EDL interference factor to characterize the above overlapping effect. Their curves for EDLs inside the slit pores are shown in Fig. 7c. The observed oscillatory behavior is similar to that of capacitance.

The above simulations are focused on the charge storage behaviors for internal EDL structures, while the external EDL structure can also affect the capacitance. As Varanasi et al.’s work [104] indicated, the curve of capacitance versus thickness of external fluid region exhibits an initial steep increase, followed by a stable region. Moreover, the addition of organic solvents affects the energy storage. On the one hand, it could slow down the oscillation of the curve of capacitance versus pore size after the first peak, resulting from modulating the polarity of solvent molecules (Fig. 7d) [105, 106]. The addition of small amount of solvent can increase the capacitance, compared with pure RTIL electrolyte [107], but a non-monotonic variation of capacitance when mole fraction increases from 0 to 1 (Fig. 7g) [108]. On the other hand, the presence of ACN molecules enhances the motions of RTIL ions (Fig. 7f) [109, 110], ascribed to the weakened Coulombic interactions caused by the longer distance between the electrolyte ions. For water solvent, it can weaken the ionic effects, leading to almost identical capacitances for diverse ions with varying diameters and valences [111]. The affinity of impurity to the nanopore can affect the capacitive performance [112].

The behavior of charge storage at different stages could be embodied in the charge storage mechanism. This mechanism can help identify the charging process at different voltages in sub-nanometer pores with varying width. In addition, the storage mechanisms can be compared when using different kinds of electrolytes. Through the analysis of the number density in the pores for the case of RTIL (Fig. 7h), Wu et al. [113] found three distinct charging stages, i.e., ion exchange for low voltage, co-ion exclusion for medium voltage and counterion adsorption for high voltage. By analyzing the capacitance curve, the damping of capacitance at high voltages is detected (Fig. 7i). This damping could be ascribed to the saturation of ions in the sub-nanometer pores [114].

With MD investigations advancing our knowledge into the EDL microstructures and mechanism of energy storage, new theoretical models are proposed for sub-nanometer pores. Feng et al. found that the ions could be located at the center of pores and form a single monolayer when the pore sizes range from 10 to 14.7 Å [25]. A new theoretical model was developed to describe the charge storage behavior for sub-nanometer pores, which agrees well with the experimental results. According to its principle, the slit-type model with single monolayer of ions can be considered as two dielectric capacitors in parallel, as illustrated in Fig. 7e.

**Fig. 8:**
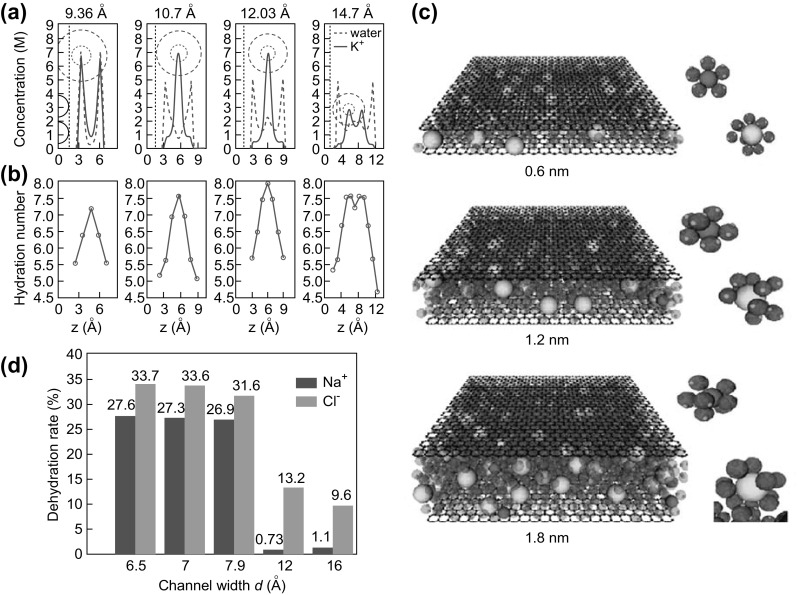
The distributions of **a** concentration and **b** hydration number as a function of position for different kinds of pore size. Reprinted with permission from Ref. [25]. Copyright (2010) American Chemical Society. **c** The hydration structures of Ca^+^ and Cl^−^ ions under the slit pores of 0.6, 1.2 and 1.8 nm. Reprinted with permission from Ref. [116]. Copyright (2009) American Chemical Society. **d** The dehydration rate of Na^+^ and Cl^−^ ions for different kinds of pore size. Reprinted with permission from Ref. [30]. Copyright (2015) Nature Publishing Group

Second, the confinement effect can promote desolvation of hydrated ions. When the solvated ions enter sub-nanometer pores, their hydration shells are partially or completely stripped, reducing their effective diameter and inducing more charges accommodated inside a single highly confined pore with a fixed volume. Therefore, the desolvation of solvated ions has a close relationship with the anomalous increase in capacitance and can be greatly enhanced by the confinement. The desolvation phenomenon can be quantified by two factors, namely its hydration number and hydration structure. Analyzing the total hydration number, Kalluri et al. [115] discovered that narrowing the pore could induce a lower hydration number when the pore sizes range from 0.9 to 1.6 nm. Additionally, local hydration number characterizes the local degree of partial desolvation. For the ions with different distances to the electrode surface, the confinement effect caused by the walls is quite different, and the ions in the pore center are less affected. Feng et al.’s work [25] demonstrated that the hydration number of K^+^ ions reaches a maximum at the position of pore center and decreases monotonously when the hydrated ions approach the electrode surface, when the pore sizes vary from 9.36 to 14.7 Å (Fig. 8b). According to these results, it is reasonable to infer that a larger pore is accompanied by a larger region with fully hydrated ions.

The hydration structure of partially dehydrated ions becomes planar inside the sub-nanometer pore. Ohba et al. [116] found a pentagonal hydration structure for Ca^+^ ions under the nanoconfinement, as shown in Fig. 8c. In addition to the planarization of hydrated ions, we can also observe the reduction in hydration numbers. Within the sub-nanometer pores, partial hydration shells of ions are stripped owing to the confinement and strong electric fields. Meanwhile, the limit of desolvation could be noted. In other words, the water molecules surrounding the ions cannot be fully removed. The planarized water molecules along the surface are free from confinement. This phenomenon can be confirmed by the constant dehydration rate of ~ 33.7%, as presented in Fig. 8d [30]. The value is precisely equal to the confined degrees of freedom divided by the total degrees.

**Fig. 9:**
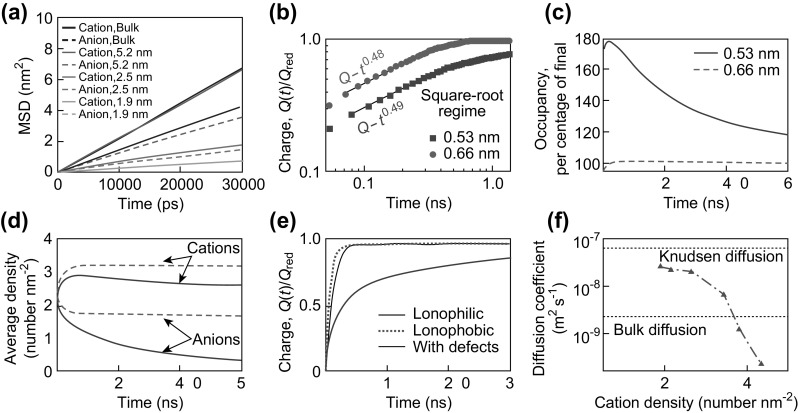
Charging dynamics and ion accumulation in slit pores. **a** MSDs of cations and anions for the slit pores with different pore sizes and bulks. Reprinted with permission from Ref. [117]. Copyright (2012) American Chemical Society. **b** The charge accumulation inside slit pores with 0.53 and 0.66 nm. When a voltage of 3 V is imposed at *t* = 0, the evolutions of **c** total pore occupancy and **d** average density of cations and anions are obtained. **e** The charge accumulation in ionophilic and ionophoboic slit pores. **f** The diffusion coefficient as a function of cation density. Reprinted with permission from Ref. [102]. Copyright (2014) Nature Publishing Group

Third, the confinement effect could weaken the dynamic characteristics of electrolyte ions. The ions in different layers suffer from different degrees of confinement, leading to distinct local dynamical behaviors with the varying distance from the ions to the wall. The dynamics of RTIL ions are weakened as they approach the walls of slit pores [117]. To demonstrate the effect of confinement on the ion dynamics, the overall dynamical behavior is introduced and compared for slit pores with different pore widths. The ions are restricted under the confinement, which can be explained by the local dynamics in different areas. With the decrease in the pore size, the region with strong local dynamics disappears preferentially, which is detrimental to the overall dynamics. A simulation work indicates that the overall dynamics of confined ions become much weakened when the confinement effect is strengthened (Fig. 9a) [117]. This observation is consistent with Salemi et al.’s work [118]. However, one simulation work indicated intensified ion dynamics with the decreasing pore size for aqueous solution [119]. It is probably attributed to some differences between the aqueous solution and the ionic liquid, including viscosity, compactness of ions, the presence of solvent molecules. Moreover, higher temperature is beneficial for the improvement in the dynamical behavior of the cations and anions inside the slit pores [119–121].

Weaker dynamics of ions could lead to a lower charging rate and longer charging time for slit pores. Kondrat et al. [102] made a comparison between a wider pore (0.66 nm) and a narrower pore (0.53 nm) and found that charging of the wider pore is faster than the narrower pore, as presented in Fig. 9b. The distinct charging rates for the wider and narrower pores are due to the different charging modes. For a narrower pore, more ions are adsorbed, while there are very few pathways for co-ions leaving the pore (mainly the process of counterion adsorption), leading to the overfilling phenomenon, as shown in Fig. 9c. The overfilling makes it easier for counterions and co-ions to leave the pore, as shown in Fig. 9d. However, the charging behavior is entirely different for a wider pore, where the major process is ion exchange, evidenced by no changes in the occupancy. The above result demonstrates that decreasing the pore size could lower the charging rate. This reduces the efficiency of EDLCs to a certain extent, despite a larger supercapacitance for narrower pores. To achieve the high energy density accompanied with a high power density inside sub-nanometer pores, Kondrat et al. attempted to strengthen the repulsion between the electrode surface and the electrolyte ions [102]. Their simulation results indicated that utilization of ionophobic pores could increase the charging rate for sub-nanometer pores, as presented in Fig. 9e. The key point of this method is to reduce the total number of ions as well as packing density inside the pores and obtain a high diffusion coefficient at the initial state, as shown in Fig. 9f.

#### Edge Effect

The edge region of graphene consists of several graphitic lines with single atom thickness, accompanied by numerous defects and dandling bonds [18, 122]. This region could promote the ion separation, leading to effective energy storage. According to experimental measurements, the specific capacitance for edge region is 4 orders of magnitude larger compared to the basal region [122]. The interpretation of this behavior could draw insights from MD simulations. According to the number of stacking layers, the edge region could be divided into edges of monolayer graphene and edges of multilayer graphene. Here we discuss the recent MD simulation work on these two kinds of edge regions.

**Fig. 10:**
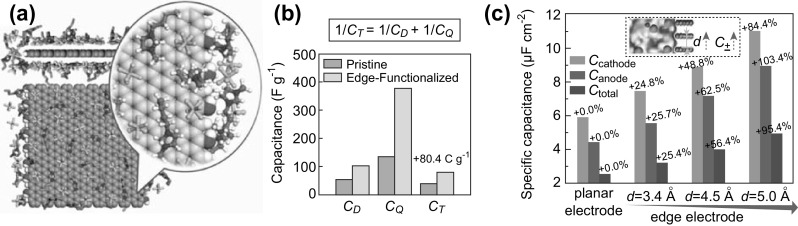
The diagrams and capacitive performances of edge. **a** The diagram of edge on graphene. **b** The capacitances of pristine and edge-functionalized graphene. Reprinted with permission from Ref. [40]. Copyright (2014) American Chemical Society. **c** The diagram of multilayer graphene and its capacitances for different intervals. Reprinted with permission from Ref. [18]. Copyright (2016) American Chemical Society

On the one hand, edges of monolayer graphene are crucial for the efficient charge storage. The simulation work on the edge effect of monolayer graphene was performed by Pak et al. [40]. This work compares the capacitances of pristine graphene and edge-functionalized one. They found higher capacitance for edge-functionalized graphene, as shown in Fig. 10b. However, the edge region was functionalized with hydroxyl group (Fig. 10a) and the interference of functional groups cannot be excluded. To illustrate the edge effect in depth, Yang et al. compared the charge accumulation for the edges and the basal planes [17]. Their results indicated a much higher charge accumulation on the edges (~ 4.2 times higher than on basal planes). The influence of edges is particularly obvious for a small length of graphene layer. It is worth mentioning that this work is mainly focused on the charge redistribution, without considering the interaction with electrolyte and the distribution of electrolyte ions. Edges could be divided into two types, namely zigzag edge and armchair edge, according to the orientation of six-membered rings. Zhan et al. utilized MD as well as DFT to clarify which type of edge is better for charge storage [123]. Their results found that the zigzag edge has a higher capacitance compared to the armchair edge, due to its very large quantum capacitance.

On the other hand, the stacks of edges are beneficial for the capacitance. Compared with monolayer graphene, stacks of graphene layers could lead to the decrease in its specific surface area, as well as capacitance. However, the stacks of edges can improve the efficiency of charge storage, even for the interlayer spacing of 0.34 nm (approaching that of graphite). Yang et al. [18] compared the capacitive performance between the planar electrode and multilayer graphene edges with different interlayer spacings, as shown in Fig. 10c. Their work showed a higher capacitance for the multilayer graphene edges than for the planar electrode (~ two fold increase at 5.0 Å). For the narrow spacing, the enhancement in capacitance is not so obvious, which could be ascribed to the poor accessibility of the interlayer by electrolyte ions. Therefore, rational control of the interval and percentage of edges could help achieve a further improvement in the energy density.

## Molecular Dynamics Investigations of 1D Nanomaterials

Carbon nanotube (CNT), a typical representative of 1D carbon material, is described as one or multiple graphitic sheets rolling up into cylindrical tubes, generally exhibiting a high length/diameter ratio [63, 124, 125]. It is known as a typical tubular morphology with the combination of excellent transport characteristics and good accessibility for its surface, making it a prominent choice for high-power systems [10, 76]. CNTs are also regarded as a suitable electrode material for flexible EDLCs. Despite these superior characteristics, its specific surface area is generally smaller than 500 m^2^ g^−1^, which restricts their broad application as high-capacitance electrodes [10]. CNTs with different numbers of rolled up graphitic sheets exhibit different efficiencies of charge storage. According to the number of graphitic sheets, CNT could be classified into single-walled carbon nanotube (SWCNT) and multi-walled carbon nanotube (MWCNT) [126]. Due to the stacks of sheets, the energy density of MWCNT is much lower than that of SWCNT. The interlayer spacing of MWCNT is approximately 0.34 nm [125], and electrolyte ions are prevented from entering the MWCNT interlayer. Therefore, the active areas for energy storage are the inner surface and the outer surface of MWCNTs, which is the same as SWCNT.

**Fig. 11:**
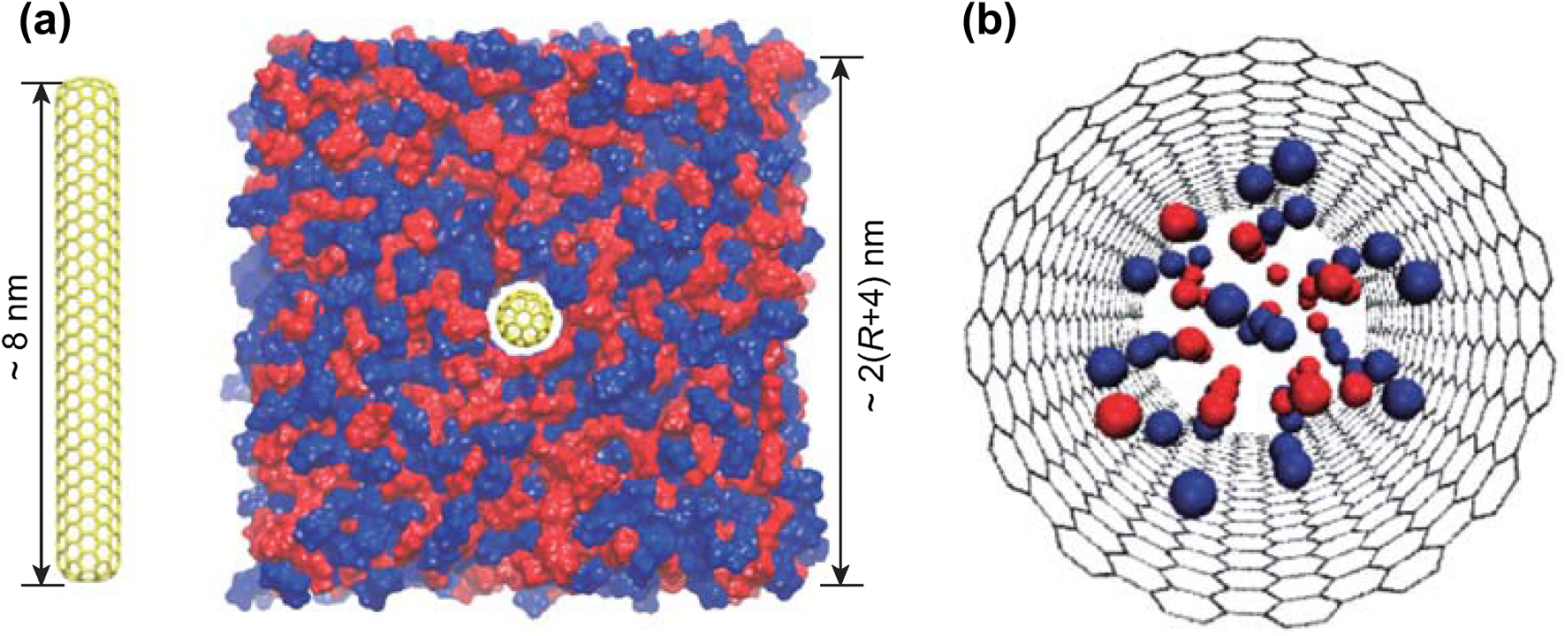
Two surfaces of CNTs. **a** The outer surface of CNTs. Reprinted with permission from Ref. [15]. Copyright (2013) American Chemical Society. **b** The inner surface of CNTs. Reprinted with permission from Ref. [28]. Copyright (2010) American Chemical Society

The surfaces of CNTs could be categorized into outer surface (Fig. 11a) and inner surface (Fig. 11b). The typical characteristics of the outer surface are the surface directly exposed to the bulk electrolyte. In this case, the confinement is much weakened or even disappears. However, a notable feature of the inner surface is that electrolyte ions can contact the surface only when the ions can access the inner region of the CNT. The major difference between the outer surface and the inner surface is the existence of confinement for the inner surface, thus leading to the numerous differences between the two types of surfaces.

**Fig. 12:**
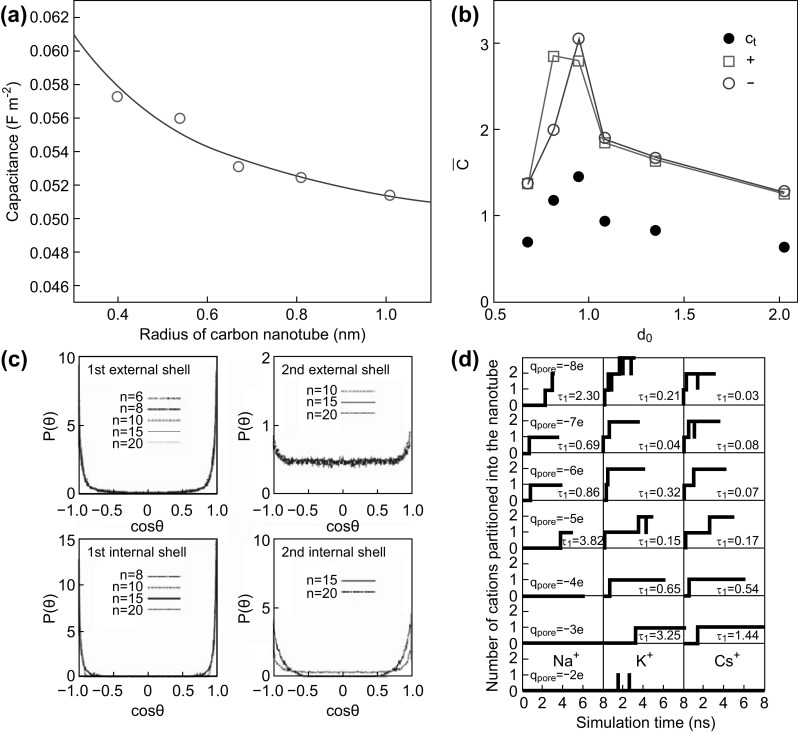
The behaviors of energy storage and microstructures for CNTs. The variation of capacitance with radius of CNT. **a** For the outer surface. Reprinted with permission from Ref. [15]. Copyright (2013) American Chemical Society. **b** For the inner surface. Reprinted with permission from Ref. [28]. Copyright (2010) American Chemical Society. **c** The orientations of ions in the first and second external shell as well as internal shell. Reprinted with permission from Ref. [16]. Copyright (2009) American Chemical Society. **d** The evolutions of number of Na^+^, K^+^ and Cs^+^ ions entering into the CNT. Reprinted with permission from Ref. [132]. Copyright (2007) American Institute of Physics

The first difference is the variation of capacitance with the radius of CNT. For the outer surface of a CNT, a monotonous increase in capacitance is observed with decreasing radius. This special behavior of charge storage is mainly ascribed to the two factors. First, when a flat plane is bent into the cylindrical surface, its outer effective area increases, so does the number of adsorbed ions. Second, without the confinement effect, the outer surface could be fully utilized. Therefore, the effective area for energy storage increases monotonously with the decrease in radius. Detailed information and further investigations should rely on MD simulations. Feng et al. [15] investigated the capacitance for a closed CNT and found that the capacitance increases continuously as the radius decreases from 1 nm to 0.4 nm (Fig. 12a). For CNT forests, a similar trend for capacitance is also observed [127]. This conclusion is confirmed for the radius larger than 1 nm, but the variation of capacitance is still unknown at sub-nanometer scale. As predicted, the variation of capacitance should qualitatively agree with Feng et al.’s results, due to the sufficient distance between the adjacent nanotubes. The inner surface is responsible for a non-monotonous increase in capacitance versus radius inside the sub-nanometer pores. Compared with the outer surface, the narrow region of the inner surface leads to the reduction in capacitance with the decrease in radius at sub-nanometer scale. This phenomenon is due to the decreasing accessibility of the inner region for electrolyte ions, leading to the reduction in the number of adsorbed ions and effective active surface area.

Shim et al. [28] employed the MD technique to study the double-layer capacitive effect for electrified CNTs with different diameters, as shown in Fig. 12b. The obtained curve of capacitance versus diameter is consistent with the experimental results [11]. For the CNT forest, the simulation work of Dive and Banerjee indicated the highest efficiency of charge storage for the interval spacing of 1.6 nm, compared to those of 1.2 and 2.0 nm [128].

The second difference is the homogeneity in orientation distribution of electrolyte ions. The orientation of electrolyte ions is the angle between artificially defined axis of ions and the normal vector of surface, and the orientation distribution is the probability distribution of the angles. For the outer surface, the electrolyte ions have a relatively isotropic orientation, compared with the inner surface. When the ions are sufficiently away from the electrode, the ions are free of confinement caused by the electrode surface and tend to show a bulk-like behavior. Shim et al. [16] investigated the orientation of ions near the electrode surface in the first and second external solvation shells. They found that nearly all EMI^+^ ions show a concentrated orientation in the first shell, i.e., parallel to the surface, but a nearly isotropic orientation in the second shell, as shown in Fig. 12c. For other solvation shells apart from first and second ones, the orientation distribution of ions is more homogeneous, due to the weakened confinement. For the inner surface, the orientation of ions is anisotropic for ions inside sub-nanometer pores. The strengthened confinement and decreasing space make the electrolyte ions tightly packed, as a consequence of the strong repulsion between the ions and the electrode. Therefore, the cations and anions have a preferable orientation distribution inside the narrow region, in order to minimize the total energy of system. For example, EMI^+^ ions in the first as well as the second internal shells are anisotropic-oriented and parallel to the CNT surface (Fig. 12c) [16]. Moreover, BMIM^+^ ions have the same orientation, as reported in Dong et al.’s work [129].

The third difference is the different dynamic behaviors of solvent molecules and electrolyte ions. For the outer surface, their dynamics are similar to the bulk-like behavior. With the increase in distance from particles to surface, the motions of solvent molecules as well as electrolyte ions resemble the motions in the bulk, due to the weakened or even disappeared confinement. The dynamics of solvent molecules and electrolyte ions are restricted inside the inner region. For solvent molecules, despite the weakened dynamic behavior under the confinement, the variations of self-diffusion coefficients with the radius are different, such as for ACN and water molecules. The dynamics of ACN molecules inside the inner region was investigated by Kalugin et al. [130], and their results indicated a monotonous increase in self-diffusion coefficient with the increase in the internal diameter. However, water molecules show a non-monotonical dependence of diffusion coefficient on the radius [131]. For electrolyte ions with different diameters, their transport into nanopores of CNTs is worth studying, which can help optimize the power density. Using three typical alkali metal ions (Na^+^, K^+^ and Cs^+^ ions) as examples, the time evolution of the ion number in the pores was investigated by Yang et al., as shown in Fig. 12d [132]. Comparing the times needed for these ions entering into the 0.67-nm nanopore, the values are in the order Na^+^ > K^+^ > Cs^+^ for the studied charge densities. For conical carbon nanopores, K^+^ ions have a faster diffusivity compared to Na^+^ ions, ascribed to the larger hydration number for K^+^ ions and the faster recovery of hydration structure [133].

## Molecular Dynamics Investigations of 0D Nanomaterials

**Fig. 13:**
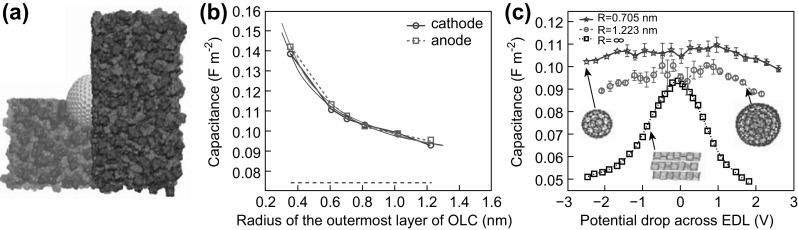
**a** The numerical structure of OLC when immersed in the electrolyte. **b** The capacitance as a function of radius of OLC. **c** The variation of capacitance versus potential for planar model and OLC model with different radii. Reprinted with permission from Ref. [135]. Copyright (2012) American Chemical Society

Onion-like carbon (OLC), a typical 0D carbon material, appears as a spherical carbon nanoparticle with several concentric one-atom-thick shells, also considered as multi-shell fullerenes and exhibiting few sub-nanometer pores [63, 76, 134]. OLC exhibits a specific area of about 500 m^2^ g^−1^, and its surface could be easily accessible to electrolyte ions, stemming from the non-porous network [58, 134]. OLC is a closed structure with its outer surface exposed to electrolyte, as shown in Fig. 13a, so the charge storage only occurs on the outer surface. Its surface also lacks the effective confinement, leading to the electrochemical performance similar to the outer surface of carbon nanotubes.

The capacitance monotonously increases with the increase in the curvature for its outer surface. In general, bending the graphitic shell to OLC can increase its effective utilization area. With stronger bending, the effective area increases, leading to the monotonous increase in capacitance. Compared with CNT, however, the effective area of OLC should be larger, because the surface of OLC is bent in four directions, while it only tends in two directions for the case of CNT. With the help of MD simulation, Feng et al. studied the capacitive effect in OLCs with the variation of radii between 0.356 and 1.223 nm to reveal its curvature effect [135]. Their simulation results indicated the increases in capacitance and accumulated ion density with the increase in its curvature (Fig. 13b). Moreover, unlike the bell or camel shapes of *C*–*V* curve for planar electrode, the variation of capacitance with voltage is very small for OLC, due to the high accessibility and short diffusion pathway for electrolyte ions. As shown in Feng et al.’s simulation work [135], *C*–*V* curve for OLC exhibits an almost flat shape, as illustrated in Fig. 13c. But the slow decrease in capacitance at high voltage indicates the appearance of saturation effect, which means that the adsorbed ions prevent the adsorption of more ions.

## Conclusion

This review examines the recent MD simulation investigations of energy storage behaviors inside the electrode materials with the structure ranging from porous to nanostructures. Few MD studies have been carried out on the AC electrodes, owing to their typical features including porosity, broad pore size distribution and the existence of numerous corrugated and curved pores. By contrast, for CDCs with relatively narrow pore size distribution, substantial MD investigations have been implemented to interpret the anomalous increase in capacitance in structures with nanoscale confinement. The typical features of porous materials enable the long diffusion pathway and relatively low power density. When the dimension of electrode materials becomes close to several nanometers, some exotic electrochemical behaviors emerge for diverse nanomaterials. Their nanoconfined space leads to their complex charging mode with the combination of ion exchange, counterion adsorption and co-ion desorption. For 2D nanomaterials, a variety of approaches have been developed to change the electrode structures and optimize the energy density, e.g., surface roughness and doping. Moreover, the nanoconfinement model based on multilayer graphene presents more details about the dynamics of energy storage. For 1D nanomaterials, their surfaces could be categorized into the inner and outer surfaces. These two surfaces have different charge storage behaviors and ion dynamics, due to the confinement effect for the inner surface. The capacitance has a monotonous increase with decreasing radius for the outer region, but the inner region leads to the reduction in capacitance with radius at sub-nanometer scale. 0D nanomaterials are the closed structures with the outer surfaces exposed to the bulk electrolyte, which lack the confinement effect, leading to the monotonous increase in capacitance.

MD simulation results could provide the guidelines for the optimization of energy density inside carbon electrodes at atomic level. The methods to enhance the capacitance could be considered from two aspects. First, the EDL capacitance can be enhanced by changing the electrode geometry. On the one hand, it is possible to rationally regulate the pore width of electrode materials to match the ion diameter with the pore width. On the other hand, one can tune the surface topography of electrode to separate cations and anions efficiently. Second, it is possible to improve quantum capacitance by electrode modification (e.g., dopant of heteroatoms and defects). Electrode modification can be used to optimize the electronic structure of the electrode materials and, consequently, the specific charge storage characteristics. To achieve high energy density for EDLCs, it is crucial to optimize the EDL capacitance and quantum capacitance simultaneously.

The methods of improving the power density of EDLCs by MD simulations can be categorized into two aspects, i.e., intensifying the ion dynamics and shortening the ion pathway. The narrow pore will restrict the dynamic characteristics of ions due to their strong adsorption on the material surface. Reduced surface affinity for ions can effectively repel the ions from the pore, leading to the strong dynamics for electrolyte ions. The morphology of electrode materials can determine the diffusion path for electrolyte ions under the charging process. To obtain the EDLCs with high power densities, one can achieve the intense ion dynamics and shorten the ion pathway by regulating the electrode structures and surface properties. Future research on enhancing the performances of EDLCs can benefit from the recommended electrode structures and surface properties provided by the MD simulations.
